# Interaction With Fungi Promotes the Accumulation of Specific Defense Molecules in Orchid Tubers and May Increase the Value of Tubers for Biotechnological and Medicinal Applications: The Case Study of Interaction Between *Dactylorhiza* sp. and *Tulasnella calospora*

**DOI:** 10.3389/fpls.2022.757852

**Published:** 2022-06-30

**Authors:** Romana Hampejsová, Miroslav Berka, Veronika Berková, Jana Jersáková, Jaroslava Domkářová, Friederike von Rundstedt, Anne Frary, Iñigo Saiz-Fernández, Břetislav Brzobohatý, Martin Černý

**Affiliations:** ^1^Potato Research Institute, Ltd., Havlíčkův Brod, Czechia; ^2^Department of Experimental Biology, Faculty of Science, Masaryk University, Brno, Czechia; ^3^Department of Molecular Biology and Radiobiology, Faculty of AgriSciences, Mendel University in Brno, Brno, Czechia; ^4^Department of Biology of Ecosystems, Faculty of Science, University of South Bohemia, České Budějovice, Czechia; ^5^W. Bock GmbH & Co. KG, Bremen, Germany; ^6^Department of Molecular Biology and Genetics, Izmir Institute of Technology, Urla, Turkey

**Keywords:** orchid tuber, biotic interaction, proteome, metabolome, lipidome, defense priming

## Abstract

Terrestrial orchids can form tubers, organs modified to store energy reserves. Tubers are an attractive source of nutrients, and salep, a flour made from dried orchid tubers, is the source of traditional beverages. Tubers also contain valuable secondary metabolites and are used in traditional medicine. The extensive harvest of wild orchids is endangering their populations in nature; however, orchids can be cultivated and tubers mass-produced. This work illustrates the importance of plant-fungus interaction in shaping the content of orchid tubers *in vitro*. Orchid plants of *Dactylorhiza* sp. grown in asymbiotic culture were inoculated with a fungal isolate from *Tulasnella calospora* group and, after 3 months of co-cultivation, tubers were analyzed. The fungus adopted the saprotrophic mode of life, but no visible differences in the morphology and biomass of the tubers were detected compared to the mock-treated plants. To elucidate the mechanisms protecting the tubers against fungal infestation, proteome, metabolome, and lipidome of tubers were analyzed. In total, 1,526, 174, and 108 proteins, metabolites, and lipids were quantified, respectively, providing a detailed snapshot of the molecular process underlying plant-microbe interaction. The observed changes at the molecular level showed that the tubers of inoculated plants accumulated significantly higher amounts of antifungal compounds, including phenolics, alkaloid Calystegine B2, and dihydrophenanthrenes. The promoted antimicrobial effects were validated by observing transient inhibition of *Phytophthora cactorum* growth. The integration of omics data highlighted the promotion of flavonoid biosynthesis, the increase in the formation of lipid droplets and associated production of oxylipins, and the accumulation of auxin in response to *T. calospora*. Taken together, these results provide the first insights into the molecular mechanisms of defense priming in orchid tubers and highlight the possible use of fungal interactors in biotechnology for the production of orchid secondary metabolites.

## Introduction

In their natural environment, plants have to interact and cope with many different organisms simultaneously at any time. Some of these interactions are beneficial and provide an advantage. For example, approximately 80% of biological nitrogen fixation is produced in symbiotic associations with bacteria. Similarly, most terrestrial plants are supported by mycorrhiza, namely the arbuscular type, formed by interaction with obligate symbiotic fungi (Schüßler et al., 2007) and, in fact, the mycorrhizal symbiosis plays an important role in plant growth and disease protection (Himaya et al., 2021). In general, mycorrhizal fungi supply plants with inorganic nutrients and water in exchange for carbohydrates. Conversely, this mutualism may occur even at an immeasurably low profit for fungi, and that is characteristic for orchidaceous mycorrhizae (Li et al., 2021). Orchids start their lives as mycoheterotrophs and depend on their fungal benefactors. Some orchids remain non-photosynthetic and fully mycoheterotrophic throughout their life cycle. Yet, even those that become photosynthetic at maturity retain their mycorrhizal fungi for protection and nutrient recovery (Schiebold et al., 2018). In natural habitats, orchids maintain a broader range of fungal partners, and the exact composition of the fungal pool is determined by the orchid species as well as the developmental stage of the plant host (Favre-Godal et al., 2020). Indeed, fungal associates can change over orchid development, and not all fungal species isolated from adult mycorrhizal roots are capable of supporting early seedling development (Meng et al., 2019).

Mycorrhizal fungi can enter at various stages of orchid life (Rasmussen, 1995). Fungal hyphae can penetrate the parenchyma cells of germinated orchid seeds, underground seedlings (called protocorms), or adult plant roots. In the adult stage of terrestrial orchids, fungal entry into roots occurs mainly through the hair tips of the roots. When hyphae penetrate root cells, the plasma membrane of the orchid cell invaginates, and the hypha becomes surrounded by a thin layer of cytoplasm. Within cells, hyphae coil into typical structures called pelotons, which greatly increase the interfacial surface area between orchid and fungus (Smith and Read, 2008). Pelotons last only a few days before they degenerate and become digested by the orchid cells. During this process, the plant cell remains functional and can be recolonized by any surviving hyphae or by fungi invading from adjacent cells. Infection by mycorrhizal fungi does not necessarily result in symbiosis and growth of an orchid. The interaction may also turn into a parasitic one or the orchid cells can reject the fungal associate (Smith and Read, 2008). The mycorrhizal fungi can overgrow orchid tissue and cause its browning and rotting, thus switching into a saprotrophic mode (Adamo et al., 2020). However, the environmental triggers and molecular mechanisms governing the switch that leads to a saprotrophic or mycorrhizal behavior in orchid mycorrhizal fungi remain unclear.

The complex nature of the orchid-fungi interaction is far from fully understood. Some reports have indicated that the symbiotic interaction does not activate orchid defense (Perotto et al., 2014). However, the antifungal enzyme endochitinase was significantly more abundant in orchids germinated with symbiotic fungi (Chen et al., 2017), indicating that orchids maintain active mechanisms that keep fungal symbionts under control and prevent extensive colonization. Plants, in general, produce a variety of secondary metabolites with antimicrobial properties and toxic proteins, including alkaloids, glucosinolates, lectins, and protein inhibitors (Santra and Banerjee, 2020; Heldt and Piechulla, 2021). Species in the Orchidaceae family are well known for producing bibenzyls and phenanthrene derivatives that regulate fungal growth (Hernández-Romero et al., 2005; Kovács et al., 2008; Cretton et al., 2018).

Terrestrial orchids can form root tubers, specialized organs that store nutrient reserves. Despite limited mechanical protection, the chemical composition of orchid tubers is a strong barrier against pathogens, and these organs rarely rot. Even though tubers have been an attractive source of nutrients and medicine throughout history (Musharof Hossain, 2011), the role and effect of biotic interactions on their composition have been largely overlooked. Here, we demonstrate that the presence of fungi has a significant impact on the orchid tuber proteome and metabolome and is critical for promoting the accumulation of bioactive compounds. The *Dactylorhiza* sp. hybrid D297 and the *Tulasnella calospora* fungus were selected for the experiments. This orchid has a high multiplication ability and was found to be suitable for both *in vitro* symbiotic and asymbiotic cultures. A fungus *T. calospora* has been isolated from many natural hosts, including *Dactylorhiza purpurella* (Hadley, 1970). It has been commonly used for symbiotic germination *in vitro* (see, e.g., Fochi et al., 2017a,b) but can also decompose its host tissue, as recently demonstrated by Adamo et al. (2020).

## Materials and Methods

### Plant Material and Growth Conditions

The *Dactylorhiza* sp. hybrid (D297) was obtained from Wolfgang Bock Pflanzenexport GmbH & Co. KG. Plants were propagated from protocorms in asymbiotic *in vitro* culture on Quoirin and Lepoivre medium with vitamins (Quoirin and Lepoivre, 1977; Valles and Boxus, 1987) enriched with activated charcoal (0.5 g·L^−1^) in a growth chamber providing 21°C and 16/8 h light/dark cycles with 100 μmol·m^−2^·s^−1^ photon flux density during light periods. Plants with developing tubers were transferred onto a tuber-growth-promoting medium (Supplementary Table S1) and cultivated for an additional 4 weeks before inoculation.

### Fungi Cultivation, Detection, and Inoculation Protocol

The mycorrhizal fungus was isolated from roots of an orchid species *Dactylorhiza majalis* (Rchb.) P. F. Hunt et Summerh. collected in the wet meadow by Koubovský pond in the South Bohemia, Czechia (48°58′49.398”N, 14°10′10.153″E). Its identity was verified by Sanger sequencing using the universal primer pair ITS1/ITS4 (White et al., 1990) by the commercial company SeqMe (Dobříš, Czechia). The isolate from family Tulasnellaceae (GenBank number OK161418) belongs phylogenetically to *Tulasnella calospora* group (Identity match: 97.5%; Jersakova and Tesitelova, unpublished data). *Tulasnella calospora* mycelium was grown at 25°C on a half-strength Murashige-Skoog medium supplemented with vitamins, 10 g·L^−1^ sucrose, and 3 g·L^−1^ agar. After 3 weeks, hyphal plugs (5 mm diameter) were transferred into 50 ml of a liquid half-strength Murashige-Skoog medium supplemented with vitamins and 10 g·L^−1^ sucrose, and mycelium was cultivated for 7 days at 50 rpm, 21°C, and 12/12 h light/dark cycles. Finally, the mycelium suspension was aliquoted, and 1 ml aliquots were used for tuber inoculation. The mock inoculation was done with 1 ml of a liquid half-strength Murashige-Skoog medium. After 3 months of co-cultivation at 21°C and 16/8 h light/dark cycles, tubers were harvested, and samples designated for omics analyses were flash-frozen in liquid nitrogen. The orchid colonization was evaluated by cross section and staining with acid fuchsin, according to Rasmussen and Whigham (Rasmussen and Whigham, 2002).

### Proteomic Analysis

At least four biological replicates (tubers of four inoculated plants and five mock-treated controls) were sampled for omics analyses. The collected samples were lyophilized and ground, and 30 mg of homogenized tissue was extracted for proteome and metabolome analyses. Total protein extracts were prepared as previously described (Hallmark et al., 2020), and portions of samples corresponding to 5 μg of peptide were analyzed by nanoflow reverse-phase liquid chromatography-mass spectrometry using a 15 cm C18 Zorbax column (Agilent), a Dionex Ultimate 3,000 RSLC nano-UPLC system, and the Orbitrap Fusion Lumos Tribrid Mass Spectrometer (Thermo Fisher). The measured spectra were recalibrated and searched against genomes of *Phalaenopsis equestris* (Cai et al., 2015), *Dendrobium catenatum* (Zhang et al., 2016), *Tulasnella calospora* AL13/4D (Kohler et al., 2015), and common contaminants. Next, all unassigned spectra were exported and analyzed by Peaks Studio 6 (BSI). The *de novo* identified peptides were searched against the UniProt database (3/2021). All matching protein sequences and peptides missing annotations were compiled into the new FASTA files and included in the final search and quantitation by Proteome Discoverer 2.5 (Thermo Fisher). The quantitative differences were determined by Minora, employing precursor ion quantification, followed by normalization and calculation of relative peptide/protein abundances. The mass spectrometry proteomics data have been deposited to the ProteomeXchange Consortium *via* the PRIDE partner repository with the dataset identifier PXD025095.

### Metabolomic and Lipidomic Analyses

Metabolites were extracted and fractionated with tert-butyl methyl ether:methanol mixture, aliquots of both fractions were derivatized and measured using a Q Exactive GC Orbitrap GC-tandem mass spectrometer and Trace 1300 Gas chromatograph (Thermo Fisher), as described in Berka et al. (2020) and Saiz-Fernández et al. (2020). Data were analyzed by Compound Discoverer 3.2 (Thermo Fisher) and searched against NIST2014, GC-Orbitrap Metabolomics library, and in-house library. Only metabolites fulfilling identification criteria (score ≥ 75 and ΔRI < 2%) were included in the final list. Dihydrophenanthrene compounds were analyzed *via* a targeted analysis of the derivatized polar fraction. Extracted chromatograms corresponding to m/z 386.1728 (Coelonin, 2TMS) and m/z 328.1489 (Orchinol, 1TMS) were analyzed employing FreeStyle 1.7 (Thermo Fisher), and fragmentation spectra were annotated with Mass Frontier 7.0 (HighChem). Coelonin standard was purchased from ChemFaces Biochemical Co., China. Samples for hormonal analyses were spiked with deuterated auxin [^2^H_5_]indole-3-acetic acid (Olchemim, CZ) and analyzed, as described in Saiz-Fernández et al. (2020). The estimation of total phenolic content was done by the Folin–Ciocalteu assay employing gallic acid as the reference (Ainsworth and Gillespie, 2007).

The lipid fraction was analyzed as previously described (Dufková et al., 2022). In brief, samples were dried by vacuum centrifugation, redissolved in 200 μl isopropanol/methanol/tert-butyl methyl ether 4/2/1 (v/v/v) with 20 mM ammonium formate, and analyzed by direct infusion employing Triversa Nanomate (Advion Biosciences) nanoelectrospray source and the Orbitrap Fusion Lumos Mass Spectrometer. The acquired profile spectra were analyzed using FreeStyle 1.7 and LipidSearch 4.2 (Thermo Fisher).

### Testing of Antimicrobial Activity

*Phytophthora cactorum* (isolate 17-37-13), obtained from the Crop Research Institute, Prague, Czechia, was propagated at 25°C on potato/dextrose broth (HiMedia) solidified with agar. The antimicrobial activity of the extract was tested by the paper disc diffusion method. In brief, 30 mg of lyophilized tubers was extracted with 1 ml of tert-butyl methyl ether:methanol mixture (3:1, v/v), and extracts were spotted on paper discs (Whatman filter paper No. 1, 6 mm diameter). For each biological replicate, a mycelial plug cut from actively growing mycelium of *P. cactorum* was placed in the center of a Petri dish and was surrounded by two sets of paper discs containing a combined amount of 140 μl of tuber extracts from inoculated and mock-treated orchids, respectively. Mycelial growth was evaluated by measuring growth distance and mycelium surface using ImageJ (Schneider et al., 2012). The whole experiment was done in three fully independent biological replicates.

### Statistical Analysis

Reported statistical tests were implemented using MetaboAnalyst 5.0 (Pang et al., 2021), SIMCA 14.1 (Sartorius), Proteome Discoverer 2.5, Compound Discoverer 3.2, STRING 11.0 (Szklarczyk et al., 2019), and MS Excel software packages. The reported quantitative differences were evaluated by Student’s *t*-test and background-based *t*-test.

## Results

### Inoculation With *Tulasnella calospora* Did Not Impact Orchid Growth and Tuber Biomass

**Figure 1:**
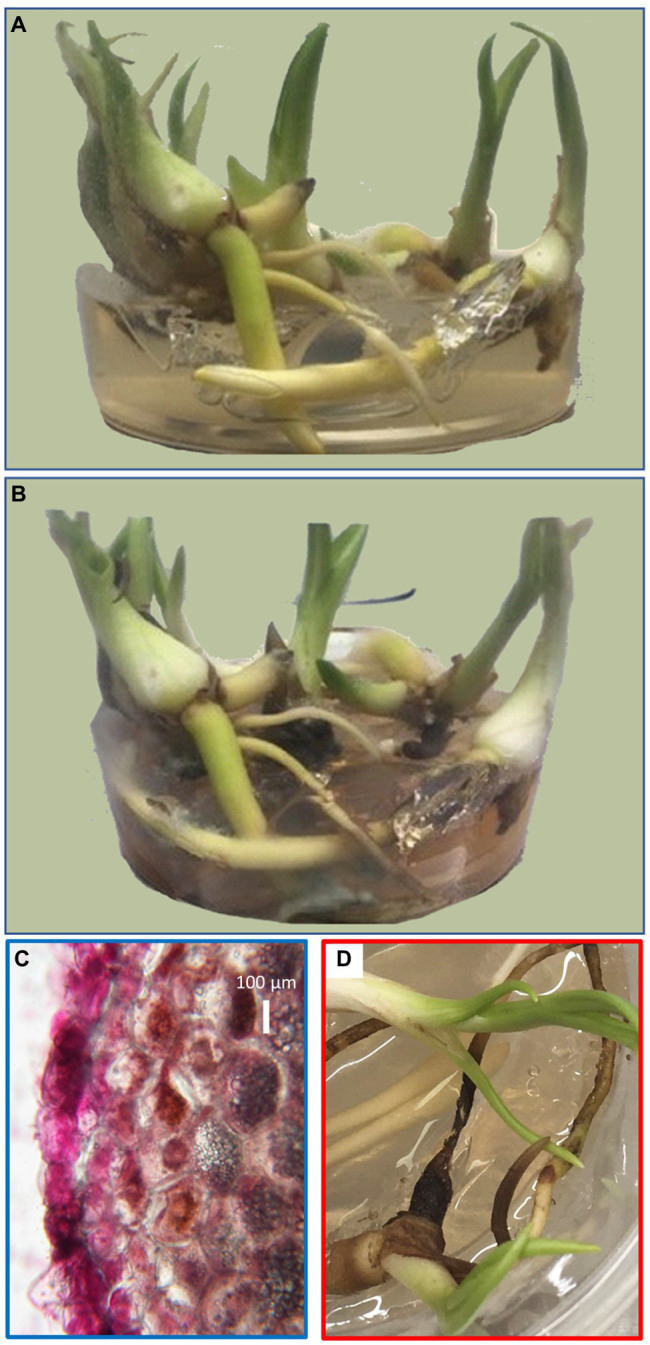
Representative biological replicate of orchid tubers before fungal inoculation **(A)** and 12 weeks after inoculation and co-cultivation with *Tulasnella calospora* **(B)**. Cross section of an orchid root with the fuchsin-stained fungi at the time of tuber sampling showing the absence of symbiotic growth **(C)** and 2 weeks later showing visible symptoms of decay **(D)**.

Orchids were grown in fully separated biological replicates in asymbiotic *in vitro* culture as described in Materials and Methods. Plants with developed tubers were then inoculated with *T. calospora* or a half-strength Murashige-Skoog medium (mock) and cultivated for an additional 3 months (Figures 1A,B). The general fitness of the orchids did not appear to be affected by fungus inoculation and no significant differences in biomass were found. However, the browning of some parts of the plant tissue indicated that the fungus had adopted the saprotrophic mode of life and the cross section of the orchid root confirmed the absence of symbiotic structures such as hyphal coils within root cells (Figure 1C). The development of tubers was not affected, the average yield was similar, and no morphological changes were detected.

### Composition of Tuber Proteome

**Figure 2:**
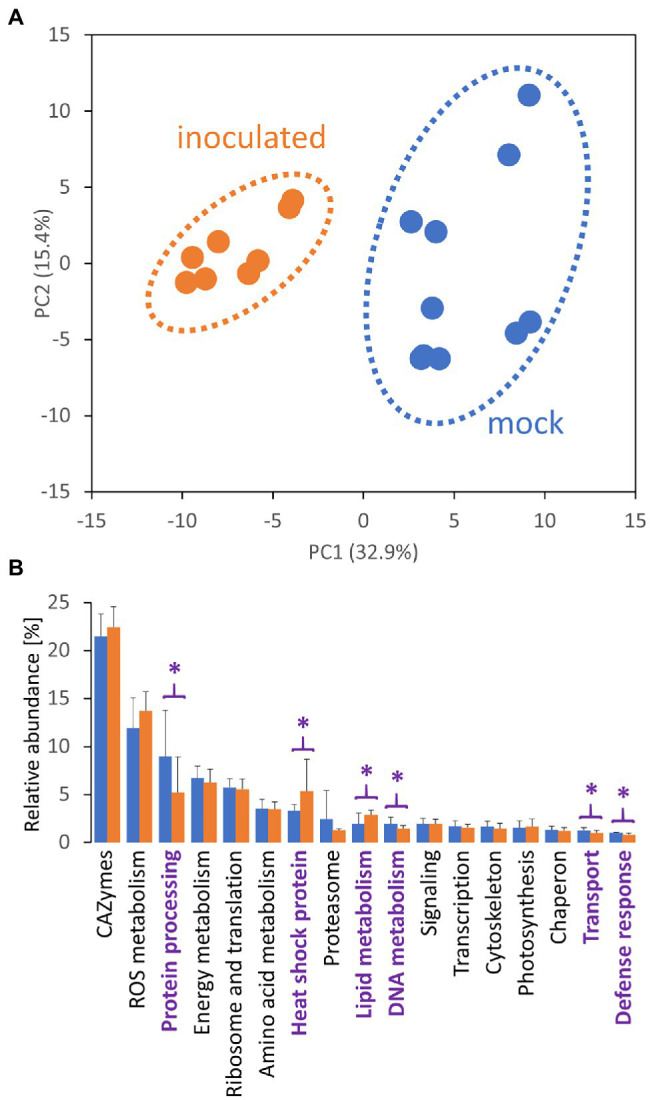
*Tulasnella calospora* inoculation modulates the composition of the tuber proteome. **(A)** Differences in proteome composition visualized by Principal Component Analysis. **(B)** Expected function and estimated relative abundances of tuber proteins. Only categories representing at least 1% of the estimated protein abundances are shown. Statistically significant differences (Student’s *t*-test; *p* < 0.05) between inoculated (orange) and mock-treated tubers (blue) are indicated (purple). Results of at least four biological replicates (tubers of four inoculated plants and five mock-treated controls) analyzed in two technical replicates. For details, see Supplementary Table S2.

Unfortunately, the genome of the selected orchid has not been sequenced. Thus, the measured spectra were searched against the genomes of related species *Phalaenopsis equestris* (Cai et al., 2015) and *Dendrobium catenatum* (Zhang et al., 2016). All unassigned spectra were analyzed by so-called *de novo* sequencing and the identified putative peptide sequences were blasted against the whole UniProt database. The final set contained 1,526 protein matches and 3,656 putative peptide sequences, and the estimated peptide abundances indicated that the identified proteins could represent less than 50% of the tuber proteome. Based on the reported data for the matching Arabidopsis orthologs, the majority of the identified tuber proteome consisted of carbohydrate acting enzymes (CAZymes) and enzymes related to ROS metabolism, proteosynthesis and protein processing, energy metabolism, and amino acid metabolism (Figure 2B).

### Effects of Inoculation on Proteome Composition

**Figure 3:**
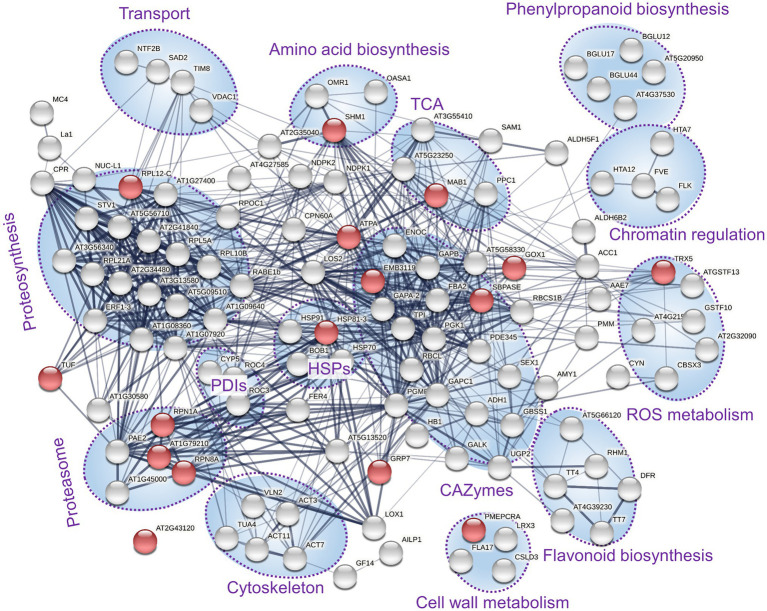
Expected role of differentially abundant proteins found in inoculated tubers. Functional enrichment of corresponding Arabidopsis orthologs and interactions visualized by String (Szklarczyk et al., 2019). The thickness of lines indicates the strength of data support; the minimum required interaction score is 0.4 (medium confidence). Red dots indicate the defense response to other organism (GO:0098542). Clusters with at least three components are highlighted. PDIs, protein disulfide isomerases; HSPs, heat shock proteins; and TCA, enzymes associated with the tricarboxylic acid cycle.

The proteome profiles clearly separated inoculated and mock-treated tuber tissues (Figure 2A). In total, we found statistically significant differences for 145 identified proteins and over 400 candidate peptides, representing around 8% of the estimated protein abundance in the mock-inoculated tubers (Supplementary Table S2). In parallel, the mycelium proteome of *T. calospora* was analyzed, and all tuber samples were searched against the *T. calospora* proteome (Kohler et al., 2015). In total, 1,498 proteins were identified in the mycelium samples (Supplementary Table S2), but the tuber proteomes did not contain any fragmentation spectra matching *T. calospora* peptides. The global comparison of inoculated tubers with mock-treated controls showed a mild but not-significant increase in the total abundance of ROS metabolism proteins and a significant increase in fatty acid metabolism, heat shock proteins, late embryogenesis abundant (LEA) proteins, and protein inhibitors. In contrast, proteins of DNA metabolism, protein processing, transport, and cell cycle were significantly depleted in the inoculated samples. Surprisingly, the summed abundance of proteins associated with biotic defense was also significantly lowered by 23%. The orthology analysis showed that the set of 145 differentially abundant proteins matched 133 Arabidopsis orthologs (Supplementary Table S2). The gene ontology functional enrichment of these Arabidopsis orthologs highlighted multiple categories (Figure 3), including “response to stress” (52 proteins), “protein metabolic process” (29 proteins), “carbohydrate metabolic process” (28 proteins), and “defense response to other organism” (16 proteins).

### Effects of Inoculation on Metabolome

**Figure 4:**
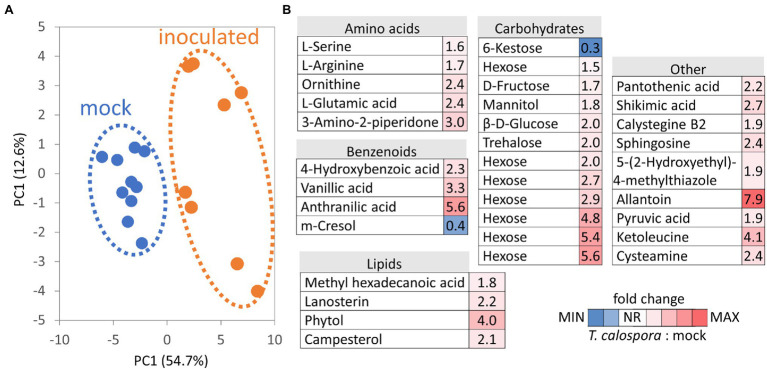
*Tulasnella calospora* inoculation modulates the pools of primary and secondary metabolites in orchid tubers. **(A)** Differences in metabolomic composition visualized by Principal Component Analysis. **(B)** Heat map visualization of detected differentially abundant metabolites. Results of at least four biological replicates (tubers of four inoculated plants and five mock-treated controls) analyzed in two technical replicates. For details, see Supplementary Table S3.

Metabolites were separated into polar and nonpolar fractions and were profiled by GC–MS untargeted analysis. In total, 174 compounds passed the detection criteria filter, 57 and 126 for nonpolar and polar fractions, respectively. Differences in metabolome profile of inoculated tubers were clearly illustrated in the PCA plot (Figure 4A), and abundances of 34 compounds in inoculated tubers were significantly different compared to controls. Only two metabolites were less abundant in inoculated tubers, namely trisaccharide identified as kestose and phenolic compound p-cresol (Figure 4B; Supplementary Table S3). The most numerous metabolite classes in the set of 32 *T. calospora*-responsive metabolites with an increase in abundance were amino acids (5) and carbohydrates and sugar alcohols (11). Metabolome pathway enrichment analysis against the Arabidopsis metabolism model highlighted a putative increase in sphingolipid metabolism (serine and sphingosine) and an enrichment in arginine and proline biosynthesis (an increase in glutamate, ornithine, and arginine abundances). Tubers of inoculated plants also accumulated metabolites and precursors of compounds with documented antifungal activity, including the phenolic compounds 4-hydroxybenzoic acid and vanillic acid, the alkaloid precursor anthranilic acid (2-aminobenzoic acid), and the metabolite annotated as tropane alkaloid calystegine B2 (Supplementary Table S3).

### Lipid Composition in Response to *Tulasnella calospora*

**Figure 5:**
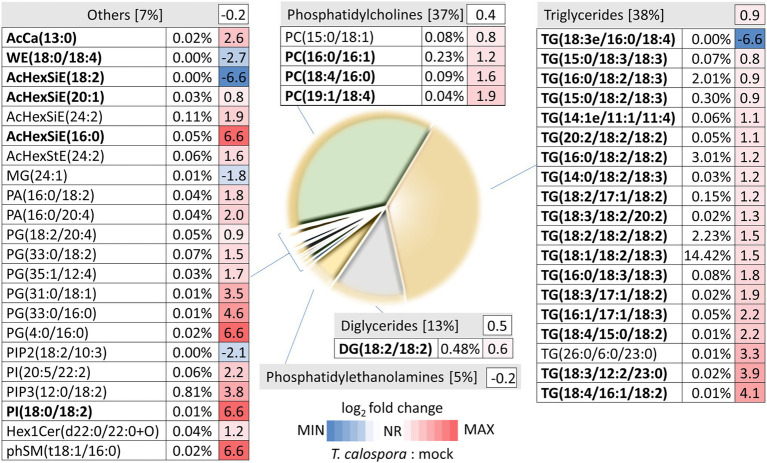
Direct infusion analysis reveals changes in lipid composition after tuber inoculation Pie chart representation of inoculated tuber lipidome and heat map visualization of the fold changes of detected differentially abundant lipids. Percentages indicate estimated relative lipid abundances based on precursor ion intensities. Data represent the means of at least four biological replicates (tubers of four inoculated plants and five mock-treated controls). AcCa, acyl carnitine; WE, wax ester; AcHexSiE, AcylGlcSitosterol ester; AcHexStE, AcylGlcStigmasterol ester; DG, diglyceride; MG, monoglyceride; TG, triglyceride; PA, phosphatidic acid; PG, phosphatidylglycerol; PC, phosphatidylcholine; PI/PIP, phosphatidylinositols; Hex1Cer, Simple Glc series; and phSM, sphingomyelin. Compounds in bold represent high-scoring identifications as determined by LipidSearch 4.2. For details, see Supplementary Table S4.

Proteome analysis revealed a significant increase in lipid metabolism (Figure 2B) and GC–MS profiling identified changes in lipid composition (Figure 4B). These results prompted a more detailed characterization of the lipid fraction. The direct infusion analyses detected more than 300 lipid species, and fragmentation spectra provided confident identification for at least 108 lipidic compounds. The most abundant compounds identified in tubers were representatives of the phosphatidylcholine, triglyceride, diglyceride, and phosphatidylethanolamine families (Figure 5). Fungi inoculation showed a statistically significant impact on 46 lipid compounds, including triglycerides, phosphatidylcholines, phosphatidylglycerols, and AcylGlcSitosterol esters (Figure 5; Supplementary Table S4). The predominant response of differentially abundant lipids was disproportionally unbalanced in favor of accumulation. In fact, only five lipid compounds were found to be significantly less abundant in tubers of inoculated plants than in controls: monoacylglycerol (24:1), AcylGlcSitosterol ester (18:2), phosphatidylinositol PIP2 (18:2/10:3), wax ester WE (18:0/18:4), and triglyceride TG (18:3/16:0/18:4). The most significant impact of inoculation was found for triglycerides. In total, 18 out of 107 identified triglycerides were significantly more abundant in inoculated tubers, contributing to a 1.9-fold increase in the total triglyceride pool. The increase in abundance appeared to be predominantly in favor of polyunsaturated triglycerides, including TG (54:6), TG (52:4), and TG (52:5), which represented more than 50% of triglycerides in tubers of inoculated plants.

### Dihydrophenanthrene Content Is Increased in Response to *Tulasnella calospora*

**Figure 6:**
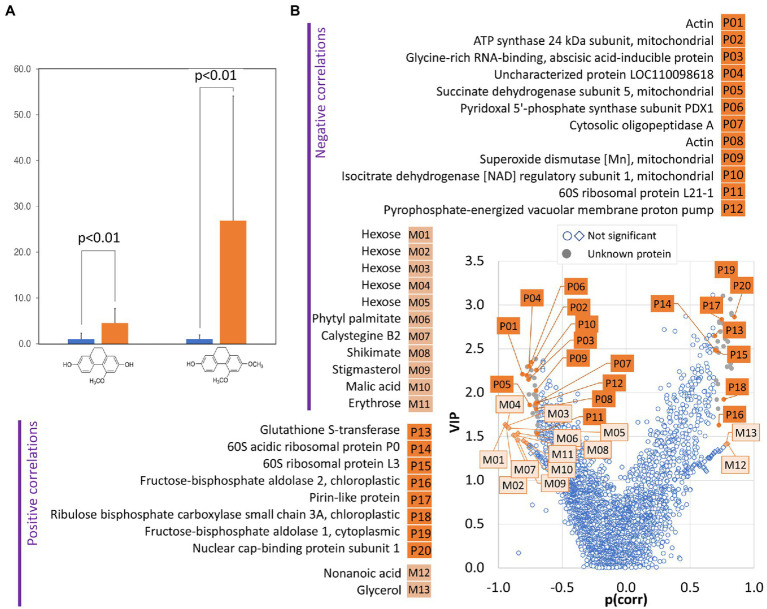
The amount of dihydrophenanthrene correlates with changes in protein and metabolites in tubers of inoculated orchids. **(A)** Relative dihydrophenanthrene content in inoculated (orange) and mock-treated (blue) orchid tubers. Data represent the means and standard deviation of at least four biological replicates (tubers of four inoculated plants and five mock-treated controls). The coelonin and orchinol content was normalized to the coelonin content in mock-treated tubers. Statistically significant differences (Student’s *t*-test) are indicated. **(B)** Identification of proteins (P) and metabolites (M) correlating with dihydrophenanthrene pool in tubers by orthogonal partial least squares discriminant analysis followed by VIP (variables of importance in projection).

Dihydrophenanthrenes are sought-after parts of salep, a flour made from the grinding of dried tubers of terrestrial orchids (Musharof Hossain, 2011). However, the untargeted metabolomics analyses did not identify any of these phytoalexins. To facilitate targeted identification, commercial salep and a dihydrophenanthrene standard were analyzed. Two metabolites matching the expected coelonin and orchinol spectra were found in tubers of the *Dactylorhiza* hybrid. The consecutive quantitative analysis showed that the dihydrophenanthrene content is significantly increased in the tubers of inoculated plants (Figure 6A).

### Tubers of Orchids Grown in Presence of *Tulasnella calospora* Manifested Accumulation of Phenolics, Substances Promoting Antimicrobial Activity, and Transient Inhibition of *Phytophthora cactorum*

**Figure 7:**
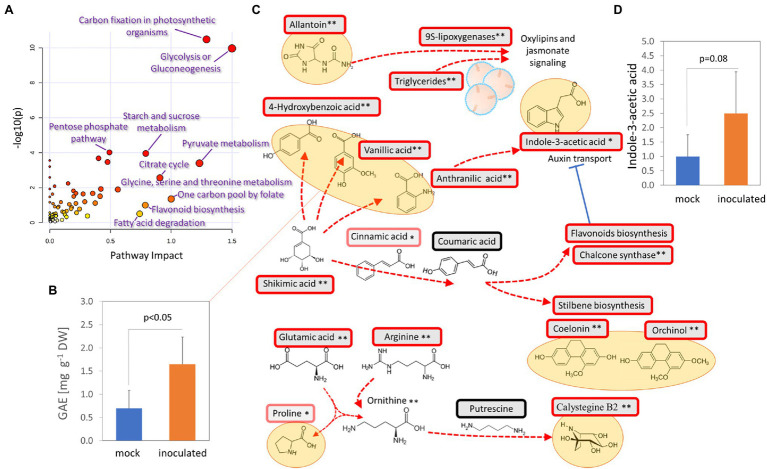
Accumulation of bioactive molecules and protectants in response to *T. calospora*. **(A)** Integration of metabolomic and proteomic data. Joint pathway analysis by MetaboAnalyst 5.0. **(B)** Estimation of total phenolic content. GAE, gallic acid equivalents. Data represent the means and standard deviation of at least four biological replicates (tubers of four inoculated plants and five mock-treated controls). **(C)** Expected metabolic flux based on integration of proteomics and metabolomics data. Statistically significant differences (Student’s *t*-test) at *p* < 0.1 (*) and *p* < 0.01 (**). **(D)** Relative auxin content in orchid tubers. Data represent the means and standard deviation of at least four biological replicates (tubers of four inoculated plants and five mock-treated controls) and were normalized to the auxin content in mock-treated tubers (48 ± 42 ng g^−1^ DW). For details, see Supplementary Tables 2 and 3.

**Figure 8:**
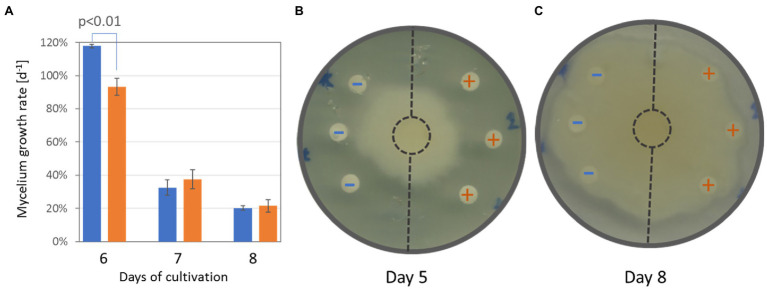
Extracts from tubers of inoculated plants show significant inhibition of *Phytophthora cactorum* growth. **(A)** The comparison of mycelium growth and **(B)** representative images of the bioassay after five and 8 days of cultivation, respectively. Data represent the means and standard deviation of three biological replicates. Blue (−)—extracts from mock-treated plants; Orange (+)—extracts from inoculated plants.

Additional experiments summarized in Figures 7A–D, 8A,B were performed to validate the results of the proteome, metabolome, and lipidome profiling. First, metabolomic analyses showed an increase in phenolic compounds. In order to validate the results of relative quantitation, the total phenolic content was estimated by the Folin–Ciocalteu assay. The results showed a 2.4-fold increase in gallic acid equivalents in tubers of inoculated orchids and confirmed the results of GC–MS metabolome profiling (Figure 7B). Next, the integration of data indicated changes in phytohormone auxin. That was confirmed, but the statistical significance was lower (Figure 7C). Finally, a test to evaluate predicted differences in antimicrobial activity was performed. Extracts from tubers of inoculated plants had a transient but significant effect on growth inhibition of *Phytophthora cactorum* (Figures 8A,B). The initial growth rate was more than 25% lower compared to extracts originating from mock-treated plants. However, the inhibition effect was lost, and the growth rates were not significantly different after 7 days of cultivation.

## Discussion

### Proteome Analysis Indicated Specialization and Reprogramming of Defense Mechanisms in Tubers of Inoculated Plants

The effect of 3-month-long co-cultivation of orchids with *T. calospora* was clearly visible as browning of portions of the root tissue, and a fraction of fine roots was fully infested with the fungi (Figures 1B,C). Our data do not provide enough evidence to determine the fungal mode of life at this stage. The assumption that the saprotrophic mode of life was activated is predominantly based on the observations collected 2 weeks after the tuber harvest (Figure 1D) and the previously reported analysis by Adamo et al. (2020). However, the fungal infestation did not manifest any significant effect on plant overall fitness. Furthermore, the morphology and size of tubers were not affected at all, and proteome analysis of these organs growing in direct contact with fungi confirmed that fungal proteins were not detectable within the tuber tissue. Orchid tubers are storage organs predominantly formed by starch and glucomannan, with an average protein content that typically represents less than 5% of the dry weight (Tekinşen and Güner, 2010; Şen et al., 2018). Proteins form an indispensable part of the plant defense system, especially those that are secreted into the apoplast or even the environment. Surprisingly, the comparison of tuber proteomes indicated that the total amount of defense-related proteins was significantly lower in the inoculated plants. The detailed pairwise comparison confirmed that putative components of defense mechanisms are significantly less abundant in the tubers of inoculated plants, including LRR family protein (putative role in effector-triggered immunity, e.g., Yoo et al., 2019), two secreted aspartyl proteases (plant-pathogen interaction; Figueiredo et al., 2021), and ricin B-like lectin (defense against pathogens; Lannoo and Van Damme, 2014). Additional orthologs of defense-related proteins such as defensin, wound-induced basic protein, and hypersensitive-induced response protein were less abundant but above the selected significance threshold (*p* < 0.05). In contrast, two pirin-like proteins and Obg-like ATPase 1 were significantly more abundant. The corresponding Arabidopsis ortholog of Obg-like ATPase is a negative regulator of disease resistance against the bacterial pathogen (Cheung et al., 2016) and plays a role in ROS production (Cheung et al., 2013). Similarly, pirin-like proteins are reportedly involved in susceptibility to bacterial plant pathogens (Zhang et al., 2014). An increase in abundance was also found for cystathionine β-synthase (CBS) domain containing protein that has a putative role in fungi resistance (Mou et al., 2015), for a putative cyanogenic β-glucosidase that may produce toxic hydrogen cyanide, and for the family of linoleate 9S-lipoxygenases (oxylipin biosynthesis and putative role in cell wall-based defense; Marcos et al., 2015). The observed changes at the proteome level indicate that the partial attenuation of defense mechanisms is a result of defense specialization induced by *T. calospora*.

Evidence of promoted defense and modulation of metabolic pathways was also found at the metabolome level. The increase in the antifungal compounds 4-hydroxybenzoic acid, vanillic acid, and anthranilic acid was likely compensated by a decrease in the abundance of cresol. Similarly, the accumulation of monosaccharides could coincide with a lower amount of kestose (Figure 4). Interestingly, a recent report indicated that a decrease in kestose is a part of the abiotic stress response (Testone et al., 2021). A role in resistance to both biotic and abiotic stresses is well established for mannitol (increased in response to *T. calospora*, Figure 4), and it serves as both an osmoprotectant and protectant against oxidative stress (Patel and Williamson, 2016). In addition to known antifungal compounds, tubers from inoculated plants accumulated compounds that can provide additional protection against *T. calospora*, including phytol (antimicrobial activity; Lima et al., 2020) and allantoin. Allantoin was the metabolite with the highest accumulation in response to *T. calospora* (7.9-fold increase, Figure 4). This compound of the purine catabolic pathway has a role in nitrogen mobilization and its accumulation is reportedly correlated with an increase in abiotic stress tolerance (Kaur et al., 2021). Arabidopsis plants treated with exogenous allantoin showed activation of jasmonate signaling (Takagi et al., 2016) that could coincide with the observed accumulation of lipoxygenases in the inoculated tubers (Supplementary Table S2). The increased abundance of ornithine, glutamate, and arginine (Figure 4) was probably also associated with the stress response. All these amino acids are elements of nitrogen metabolism, as well as precursors of the stress response metabolites polyamines and proline (Anwar et al., 2018). In fact, the proline pool was 1.4-fold higher in inoculated tubers, albeit at a lower statistical significance level (*p* = 0.1; Supplementary Table S3). On the contrary, the abundance of putrescine, the only detectable polyamine, did not change in tubers of inoculated plants (Supplementary Table S3). However, putrescine is a direct precursor of tropane alkaloids, and it is thus likely that it was metabolized to calystegine B2 (Dräger, 2004). Finally, functional enrichment highlighted an increase in flavonoid biosynthesis (Figure 3), and the impact of this metabolic pathway was confirmed by the integration of metabolic and proteomic data (Figure 7A). The increase in the abundance of shikimic acid (Figure 4), cinnamic acid (3.2-fold, *p* = 0.1; Supplementary Table S3), and a key enzyme of flavonoid biosynthesis (chalcone synthase TT4, *p* < 0.05; Supplementary Table S2) indicates a positive modulation of flavonoid biosynthesis in inoculated tubers. Flavonoids are integral compounds of plant-microbe interaction (e.g., Shah and Smith, 2020), and the detected depletion of dihydroflavonol reductase, isoflavone reductase, and flavonoid 3′-monooxygenase (Supplementary Table S2) showed that the *T. calospora* presence attenuated some branches of flavonoid biosynthesis.

The observed effects are well-in-line with known responses to fungal effectors and effector-triggered immunity (e.g., Selin et al., 2016). We have not identified any *T. calospora* proteins found in its mycelium, but we cannot exclude that fungal effectors that are not present in the mycelium escaped our identification. However, none of the identified proteins exclusively found in tubers of inoculated orchids match a putative effector, indicating that a systemic response is a more likely explanation.

### Triglyceride Accumulation Indicates the Activation of Lipid-Droplet-Based Oxylipin Production

Lipids and lipid-derived molecules play an important role in plant-microbe interactions (Siebers et al., 2016; Mehta et al., 2021). Lipidome profiling revealed a significant increase in triglycerides. These compounds, together with other lipids and coating proteins, form the basis for lipid droplets, universal cellular organelles that act as storage compartments of lipids. Triglycerides formed more than 20% of the estimated lipid content in the mock-treated orchid tubers, and that percentage nearly doubled in response to *T. calospora* inoculation (Figure 5). Lipid droplets in Arabidopsis provide a source for the production of oxylipins derived from polyunsaturated fatty acids and play a role in response to fungus infections (Shimada et al., 2014). It seems that the same process was triggered in orchid tubers, and the accumulation of triglycerides and a higher proportion of polyunsaturated triglycerides (Figure 5) is part of the defense response. That is well in line with the observed accumulation of lipoxygenases, and the triglyceride accumulation probably coincides with the increased abundance of acylcarnitine (associated with anabolic processes of lipid metabolism; Nguyen et al., 2016). Furthermore, the formation or expansion of lipid droplets could explain the observed increase in the phosphatidylcholines required for droplet coating (Krahmer et al., 2011). Finally, it is tempting to speculate that the accumulation of campesterol (a precursor of brassinosteroids; Figure 4) and phosphatidylinositols (Figure 5) is a direct evidence of promoted defense signaling (Hung et al., 2014; Hussain et al., 2020).

### Correlation of Dihydrophenanthrene Content With Tuber Composition

The targeted analysis showed that the dihydrophenanthrene compounds identified as coelonin and orchinol were significantly more abundant in inoculated tubers. However, the biological variability was high. The production of phytoalexins is an indirect marker of biotic interaction and the observed variability led us to perform an orthogonal partial least squares (OPLS) regression analysis and evaluate the correlation between the dihydrophenanthrene content and the tuber composition. As illustrated in Figure 6B, 20 proteins and 13 metabolites showed a strong correlation with the dihydrophenanthrene pool. Nine of these proteins were identical with the list of identified differentially abundant proteins (Supplementary Table S2), including putative components of biotic interactions and defense mechanisms (pirin-like protein and glutathione S-transferase), and the OPLS provided supporting evidence for the role of additional proteins in the observed biotic interaction. Proteins such as the nuclear cap-binding protein subunit (involved in RNA splicing and RNA-mediated gene silencing) and organellar oligopeptidase A (modulates salicylic acid signaling and the immune response; Moreau et al., 2013) did not pass the significance threshold of pairwise comparison (1.5-fold change, *p* < 0.05) but showed significant correlation with the dihydrophenanthrene accumulation. Interestingly, a negative correlation with dihydrophenanthrenes was found for compounds that accumulated in response to *T. calospora*, including shikimic acid and Calystegine B2. Shikimic acid is a precursor of stilbene and dihydrophenanthrenes, and its negative correlation likely represents this metabolic flow. In contrast, calystegines originate from the polyamine biosynthetic pathway, and the negative correlation with the dihydrophenanthrene pool probably coincides with a shift in the defense molecule biosynthesis. In general, calystegines are inhibitors of glycosidases (Molyneux et al., 1993), enzymes that are secreted by pathogenic fungi to facilitate plant cell wall degradation and entry into the host (Rafiei et al., 2021). In light of the observed accumulation patterns (calystegine, dihydrophenanthrenes, and putative mediator of salicylic acid signaling), it is tempting to speculate that calystegines and dihydrophenanthrenes represent targets of jasmonic acid and salicylic acid signaling, respectively. These two hormonal pathways are usually antagonistic, though the interaction is far from simple (e.g., Moreira et al., 2018).

### *Tulasnella calospora* Inoculation Modulates Auxin Pool in Orchid Tubers

A strong negative correlation with the dihydrophenanthrene content was found for an ortholog of the Arabidopsis pyrophosphate-energized vacuolar membrane proton pump 1 (Figure 6B), which is involved in the control of auxin transport (Li, 2005). In parallel, an ortholog of small heat shock protein BOB1 required for the establishment of auxin gradients (Jurkuta et al., 2009) was significantly less abundant in response to the inoculation (Supplementary Table S2), and the comparison of the list of differentially abundant proteins with previously identified phytohormone-responsive proteins (Černý et al., 2016) revealed 16 putative auxin-responsive proteins. Interestingly, auxin induces actin filament unbundling (Arieti and Staiger, 2020), and the set of identified differentially abundant proteins contained five components of the cytoskeleton (Supplementary Table S2), including an ortholog of the actin-binding protein Villin-2 (significantly less abundant in response to inoculation) involved in the bundling of actin filaments. The subsequent analysis of the indole-3-acetic acid pool showed a positive correlation with the dihydrophenanthrenes (Pearson’s correlation coefficients *r* > 0.8) and a 2.5-fold increase in inoculated tubers compared to controls, but the statistical significance of the pairwise comparison was lower (*p* < 0.1; Figure 7D). Auxin precursor anthranilic acid and enzyme nitrilase (NIT) were significantly more abundant in tubers of inoculated plants (Supplementary Table S2). Arabidopsis NIT1 (60% identity) is an auxin biosynthetic enzyme, but the sequence alignment indicated that this isoform is most likely orthologous to NIT4 (72% identity) and does not possess auxin biosynthetic activity. The observed accumulation of auxin could coincide with an increase in the abundance of the flavonoid biosynthetic enzyme chalcone synthase TT4. Flavonoids act as endogenous negative regulators of auxin transport (Brown et al., 2001), and the inhibition of auxin transport could be a side effect of stress-induced flavonoid biosynthesis. Taken together, these results indicate that *T. calospora* is affecting auxin metabolism and transport, resulting in its accumulation in tubers of inoculated plants (Figures 7C,D).

### *Tulasnella calospora* Increases Antimicrobial Potential of Tubers

Interactions with microbes stimulate plants in different ways. Plant-associated microorganisms impact the plant’s growth, accumulation of secondary metabolites, and, consecutively, the resistance to biotic and abiotic stressors (Fontana et al., 2021; French et al., 2021; Jahn et al., 2022). The accumulated secondary metabolites originate both from microbes and the microbe-activated plant metabolism, and it has been shown that a co-culture of plants with endophytic fungi could significantly enhance the production of valuable plant bioactive compounds (e.g., Ding et al., 2018; Ye et al., 2021). Here, the orchid tubers of inoculated plants accumulated multiple compounds with known antimicrobial activity. The most significant difference was found for dihydrophenanthrenes that are phytoalexins shown to be active against *Phytophthora* (Ward, 1975). In order to test the tuber extract activity, the mycelium of *P. cactorum* was grown in the presence of tuber extracts from inoculated and mock-treated plants. *Phytophthora* growth was not arrested. However, the comparison showed that the mycelium growth in the direction of extracts from inoculated plants was significantly and reproducibly inhibited (Figures 8A,B). That inhibition was only transient, indicating that the amount of extracted inhibitors was not sufficient or that the inhibitors were metabolized by *Phytophthora*.

## Conclusion

Mycorrhizal symbionts provide essential nutrients for orchid germination and early development. However, the same beneficial fungus eventually becomes less profitable, and the plant may try to suppress the growth of its former benefactor. This work provided the first insight into the molecular composition of orchid tubers in response to fungi with an apparent saprotrophic mode of life. There were no visible differences in tuber morphology, but the observed changes at proteome, metabolome, and lipidome levels showed that tubers of inoculated plants had activated specialized defensive pathways and harbored significantly higher amounts of antifungal compounds compared to that of seemingly similar tubers of mock-treated plants. Integrative results underlined the molecular processes that protect tuber tissue from fungal infestation and illustrated the importance of fungus in the accumulation of secondary metabolites. The results of our experiments cannot be generalized and extrapolated to all orchid-fungi interactions. However, orchids have been and are being used as medicine, and these results indicate that the absence of fungal interactors may significantly decrease the pharmacological value of orchid products.

## Data Availability Statement

The datasets presented in this study can be found in online repositories. The names of the repository/repositories and accession number(s) can be found at: https://www.ebi.ac.uk/pride/archive/, PXD025095.

## Author Contributions

MC and RH designed research. RH prepared all physiological experiments and contributed to the manuscript draft. MB, MC, and VB performed metabolomics analyses. MC performed proteome and lipidome analyses and analyzed omics data. MC, MB, JD, JJ, FR, AF, IS-F, and BB reviewed and analyzed results. BB and JD raised funding. MC prepared figures and wrote the manuscript. All authors contributed to the article and approved the submitted version.

## Funding

Funding support for this work was provided by the Ministry of Education, Youth and Sports of the Czech Republic (CR) to MC, MB, VB, IS-F, and BB (grant no. CZ.02.1.01/0.0/0.0/16_019/0000738), with support from the European Regional Development Fund—Project “Centre for Experimental Plant Biology,” the Ministry of Education, Youth and Sports of the Czech Republic (CR) to RH, JD, and FR (EUREKA grant no. LTE218006; Ʃ! 12210), and the Internal Grant Agency of Mendel University in Brno grant AF-IGA2022-IP-049 to VB.

## Conflict of Interest

RH and JD were employed by Potato Research Institute, Ltd., Havlíčkův Brod, Czechia. FR was employed by W. Bock GmbH & Co. KG, Bremen, Germany.

The remaining authors declare that the research was conducted in the absence of any commercial or financial relationships that could be construed as a potential conflict of interest.

## Publisher’s Note

All claims expressed in this article are solely those of the authors and do not necessarily represent those of their affiliated organizations, or those of the publisher, the editors and the reviewers. Any product that may be evaluated in this article, or claim that may be made by its manufacturer, is not guaranteed or endorsed by the publisher.
